# Defective angles of localized retinal nerve fiber layer reflect the severity of visual field defect- a cross-sectional analysis

**DOI:** 10.1186/s12886-020-01396-y

**Published:** 2020-04-09

**Authors:** Alexander Chen, Ing-Chou Lai, Wan-Hua Cho, Hung-Yin Lai, Pei-Wen Lin, Pei-Chang Wu, Ming-Tse Kuo

**Affiliations:** 1 Department of Ophthalmology, Kaohsiung Chang Gung Memorial Hospital and Chang Gung University College of Medicine, Kaohsiung, 83301 Taiwan; 2 Chang Gung University College of Medicine, Taoyuan, 333 Taiwan

**Keywords:** Localized retinal nerve fiber layer, Visual field defect parameters, Fundus photograph

## Abstract

**Background:**

In order to detect glaucomatous optic nerve damages early on and evaluate the severity of glaucoma, a previously developed analytic method based on photographic retinal nerve fiber layer (RNFL) angle defect was proposed. However, the correlation between these defective angles and the severity of visual field defect has not been verified. This study aimed to confirm the correlation described above.

**Methods:**

We reviewed a total of 227 glaucomatous eyes (38 enrolled, 189 excluded) during an interval of 5 years. The angles of all eyes were measured on RNFL photograph, of which angle α is the angular width between the macula center and the proximity of RNFL defect, and angle β (+c) is the sum of angular width(s) of localized RNFL defect. The severity of visual field defect was determined by mean deviation (MD), pattern standard deviation (PSD), and visual field index (VFI). Correlation analysis was performed on angle α and angle β (+c) with the presence of central scotoma and visual field defect parameters, respectively.

**Results:**

Angle β (+c) showed significant correlation with MD (*P* = 0.007), PSD (*P* = 0.02), VFI (*P* = 0.03), and average RNFL thickness (*P* = 0.03). No correlation was found between angle α and the presence of central scotoma.

**Conclusions:**

In conclusion, measuring the angular width of localized RNFL defect is a viable method for determining the severity of visual field defect.

## Background

Glaucoma is globally ranked the second most common cause of blindness [1]. Early detection is of utmost importance for glaucomatous eyes, and using conventional retinal nerve fiber layer (RNFL) photograph to evaluate localized retinal nerve fiber layer defects (RNLFD) is a tool of choice for detecting early glaucomatous eyes when optical coherence topography (OCT) is unavailable. However, there is a lack of established quantitative analysis using RNFL photograph.

Woo et al. previously established a convenient quantitative method for analyzing localized RNFL defect using RNFL photograph by measuring the angles around the disc [2]. They first defined the reference line as the line between the macula center and the optic disc center. Angle α is the angular width between the reference line and the proximity of RNFL defect, while angle β (+c) is the sum of angular width(s) of localized RNFL defect. This method was then used to compare different etiologies of various types of glaucoma [2, 3]. The idea was built upon the assumption that localized RNFLD or the optic nerve head configuration [4–7] is somehow correlated with the visual field defect, but the validity of such correlation has not been verified.

There are several implications in validating such quantitative method. Firstly, if the sum of the angular width of the localized RNFL defect, or angle β (+c), is indeed correlated with visual field defect, the severity of visual field defect can be predicted by the morphological defect of localized RNFL. Secondly, if the angular width between the reference line and the proximity of RNFL defect, or angle α, is correlated with central scotoma, we may use this as an indicator for earlier or more aggressive treatment [8] since central visual field defect drastically affects patient’s life quality [9]. In fact, patients with central visual field defect are associated with reading difficulty [10], worsening of driving performance [11], and are at greater risk of visual acuity loss [12]. The verified method can possibly be adopted as a new parameter in optical coherence tomography (OCT). Moreover, this method can be popularly implemented in local clinics and developing countries without OCT.

Thus, this study aims to confirm the correlation between the localized RNFL angle defect and the visual field defect.

## Methods

### Participant recruitment

**Fig. 1:**
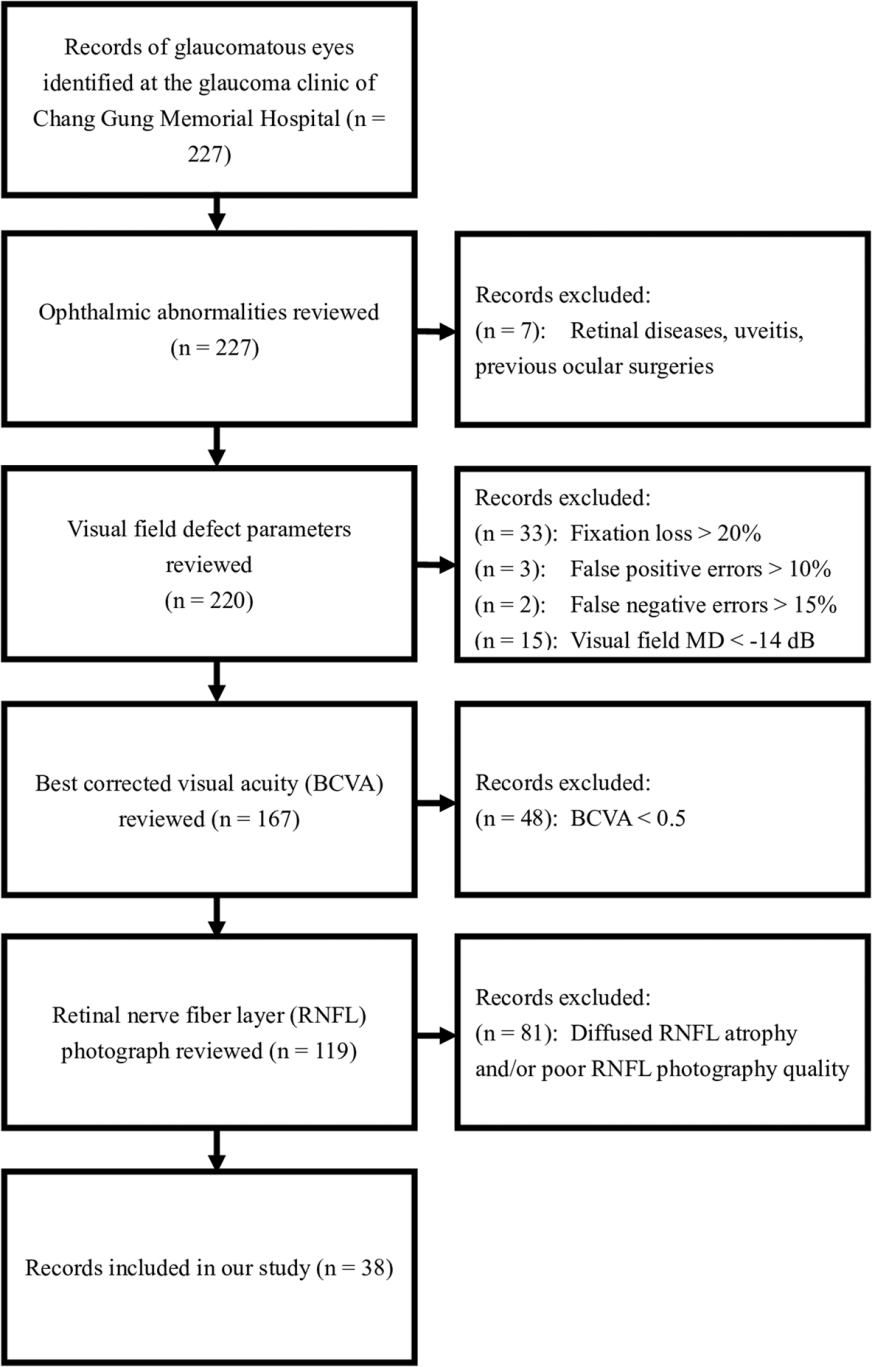
Inclusion and exclusion flowchart. A total of 227 clinical records were reviewed. After scrupulous screening using the above exclusion criteria, 38 eyes were included into our study

This study was approved by the Institutional Review Board of Chang Gung Memorial Hospital, Kaohsiung, Taiwan (Registration Number: 201701398B0) and adhered to the tenets of the Declaration of Helsinki. A retrospective single-center cross-sectional study of glaucomatous eyes was conducted. The clinical records of 227 glaucomatous eyes (38 enrolled, 189 excluded) diagnosed at the glaucoma clinic of Chang Gung Memorial Hospital during an interval of 5 years were carefully reviewed and were anonymized before analysis (Fig. 1).

All patients underwent complete ophthalmological evaluations including Snellen visual acuity measurements, gonioscopy, pneuma-tonometry, dilated fundus examination of the optic disc with a 90-diopter lens (Volk super 666, Volk Optical Inc., Mentor, Ohio, USA), RNFL photography (Topcon retinal camera TRC-50EX, Itabashi-ku, Tokyo, Japan), and visual field test with Humphrey field analyzer (30–2 Program, Carl Zeiss Meditec, Inc., Dublin, CA, USA). Average RNFL thickness was obtained from spectral domain optical coherence tomography (Spectralis OCT, Heidelberg Engineering, Heidelberg, Germany) for comparison since previous studies have reported correlation between visual field defect and average RNFL thickness [13–15].

Glaucomatous eyes were defined using the Anderson-Patella’s criteria. Visual field defects had to be compatible with RNFL defects and repeatable on at least two consecutive tests. The exclusion criteria included those with previous ophthalmic abnormalities such as retinal diseases, uveitis, previous ocular surgeries, best-corrected visual acuity (BCVA) < 20/50, as well as cases that were difficult to identify RNFL defects due to poor quality of RNFL photograph (Fig. 1).

### Visual field defect parameters

The parameters of visual field defect severity were determined by visual field mean deviation (MD), visual field pattern standard deviation (PSD), and visual field index (VFI). No previous studies defined central scotoma with 30–2 visual field test, so we proposed the following criteria to define central scotoma: two or more points with < 5% and/or one or more points with < 2% within the central 6 degrees of fixation. In order to reinforce the reliability of our visual field parameters, we excluded those with fixation losses > 20% [16], false positive errors > 10%, false negative errors > 15%, and visual field MD < − 14 dB (Fig. 1).

### Localized retinal nerve fiber layer defect parameters

**Fig. 2:**
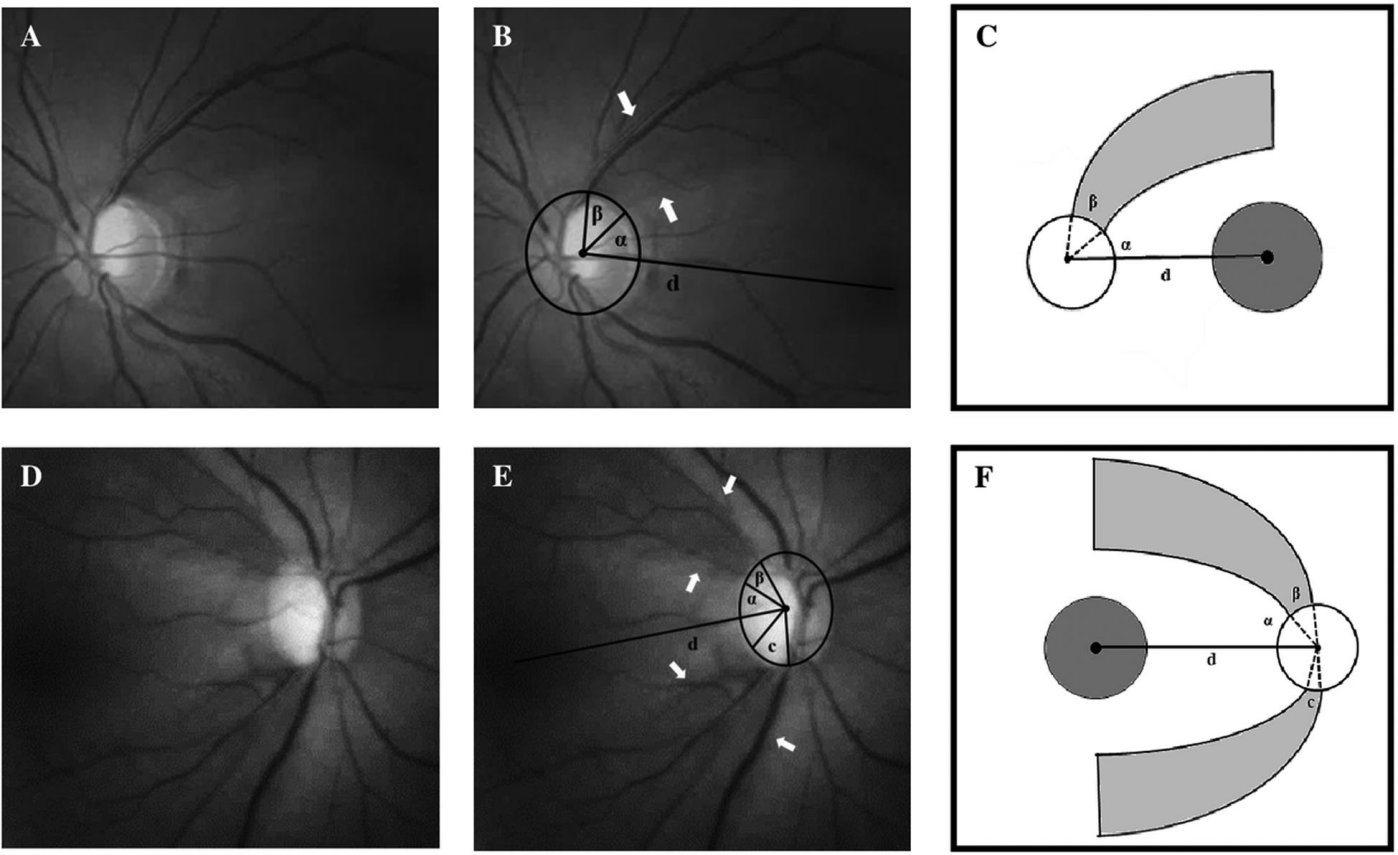
Parameters of localized retinal nerve fiber layer (RNFL) defect were identified using RNFL photography. **a** Localized RNFL defect of a left eye without labeling. **b** Localized RNFL defect with labeled parameters. **c** Schematic illustration of (**b)**. **d** Localized RNFL defect of a right eye without labeling. **e** Localized RNFL defect with labeled parameters. **f** Schematic illustration of (**e**). Localized RNFL defect is defined with the white arrows. Reference line d is the line between the macula center and the optic disc center. Angle α is the angular width between the reference line and the proximity of the RNFL defect. Angle β (+c) is the sum of angular width(s) of localized RNFL defect

Localized RNFL defect was defined as wedge-shaped non-spindle-like defects touching or running towards the optic disc border [17]. The method [2] of angle measurements for evaluating localized RNFL defect was as follows (Fig. 2):

#### Reference line = the line between the macula center and the optic disc center

1. Angle α = the angular width between the reference line and the proximity of RNFL defect.
2. Angle β (+c) = the sum of angular width(s) of localized RNFL defect.

Those with localized RNFL defects greater than 120 degrees from the reference line were excluded since the nasal RNFL defects were hard to determine. The average degrees of defective angles in RNFL photographs, of which patients’ medical record information were blinded, were measured using ImageJ software (version 1.51j8, developed by Wayne Rasband, National Institutes of Health, Bethesda, MD, USA http://imagej.nih.gov/ij) by three ophthalmologists. We performed an analysis on inter-rater reliability using SPSS to evaluate the absolute agreement between the raters and found that the correlation of the average measures of angle α and angle β (+c) among the three raters was 0.998 (95% confidence interval 0.996–0.999) and 0.996 (95% confidence interval 0.992–0.998), suggesting very strong correlations among the three raters.

Correlation analysis was then performed on angle α and angle β (+c) with the presence of central scotoma and visual field defect parameters, respectively.

### Sample size determination

Using a free power calculator (http://powerandsamplesize.com/Calculators/Validations), the sample size of at least 37 eyes was estimated based on the mean differences of angle α and angle β (+c) per 1 dB change of MD between high and low myopia patients according to Kim et al. under the power of 0.90 [3].

### Statistical analysis

All statistical analyses were performed using SPSS software (version 12.0; SPSS Inc., Chicago, IL, USA). Data were presented in the forms of means ± standard deviation or frequencies unless otherwise specified. A *P* value < 0.05 was considered as statistically significant. Spearman’s rank correlation test was used to analyze the association of angle α and the presence of central scotoma as well as angle β (+c) and visual field defect parameters. Inter-rater reliability was performed using two-way mixed and absolute agreement settings.

## Results

A total of 38 glaucomatous eyes of 227 patients were enrolled for this cross-sectional analysis with the mean age of 59.0 ± 8.8 years. Baseline characteristics were listed in Table 1. Participants were of early glaucoma defects with an average visual field MD of − 4.7 ± 3.2 dB and an average RNFL thickness of 76.1 ± 16.3 μm. The mean of angle α and angle β (+c) was 41.1 ± 17.2 and 53.8 ± 20.4. Spearman’s rank correlation test was used to analyze the correlation of angle α with the presence of central scotoma (*P* = 0.82) and average total macular thickness (*P* = 0.21) and no correlations were found. In Table 2, angle β (+c) was significantly correlated with MD (*P* = 0.007), PSD (*P* = 0.02), VFI (*P* = 0.03), and average RNFL thickness (*P* = 0.03).

**Table 1:** Baseline characteristics of the participants

	Glaucomatous eye
Right eyes	17 (44.7)
Age (yr)	59.0 (8.8)
Male	22 (57.9)
Spherical Equivalence (D)	−0.5 (2.6)
Angle α (degrees)	41.1 (17.2)
Angle β + c (degrees)	53.8 (20.4)
Intraocular Pressure (mmHg)	14.8 (4.1)
**Humphrey field analyzer 30–2**	
Visual Field Index (%)	90.0 (9.8)
Fixation Losses	5.7 (6.2)
False Positive Errors (%)	1.4 (1.7)
False Negative Errors (%)	2.1 (2.9)
Visual Field MD (dB)	−4.7 (3.2)
Visual Field PSD (dB)	6.1 (4.4)
Presence of Central Scotoma	26 (68.4)
**Spectralis optical coherence tomography**	
Average RNFL Thickness (μm)	76.1 (16.3)
Average total macular thickness (μm)	269.3 (16.4)

Values are expressed as number (frequency) or average (standard deviation)

*MD* mean deviation, *PSD* pattern standard deviation, *RNFL* retinal nerve fiber layer

**Table 2:** Correlation between localized retinal nerve fiber layer defect angle β (+c) and visual field defect parameters, and average retinal nerve fiber layer thickness

	Correlation	MD	PSD	VFI	Average RNFLT
Angle β + c	R (95% CI)	**−0.428 (−1.17–0.73)**	**0.366 (− 0.45–1.39)**	**−0.364 (− 0.79–1.69)**	**−0.350 (− 0.65–0.216)**
	*P*	**0.007**	**0.02**	**0.03**	**0.03**

*CI* confidence interval, *MD* mean deviation of visual field, *PSD* pattern standard deviation of visual field, *VFI* visual field index, *RNFLT* retinal nerve fiber layer thickness, *P P* value, *R* Spearman’s correlation coefficient

Spearman’s rank correlation was performed

Correlations significant at ***P*** **< 0.05 are bolded**

As for the angular measurements of RNLFD relative to the sectoral RNFL thickness, Spearman’s rank correlation test was used to analyze their correlation and found that they were negative correlated significantly (*P* = 0.01). Further analysis on the correlation between OCT sectoral RNFL defect and the severity of visual field defect (Table 3) showed no significant correlation with MD (*P* = 0.34), PSD (*P* = 0.41), and VFI (*P* = 0.14). We then analyzed the correlation of global (average) RNFL thickness with the visual field parameters (Table 2) and found that average RNFL thickness was significantly correlated with MD (*P* = 0.000), PSD (*P* = 0.003), and VFI (*P* = 0.001).

**Table 3:** Correlation among visual field defect parameters, localized retinal nerve fiber layer defect angle β (+c), sectoral, and average retinal nerve fiber layer thickness

	Correlation	MD	PSD	VFI
Sectoral	R	0.161	−0.136	0.245
RNFLT	*P*	0.34	0.41	0.14
Average	R	**0.659**	**−0.469**	**0.510**
RNFLT	*P*	**0.000**	**0.003**	**0.001**

*MD* mean deviation of visual field, *PSD* pattern standard deviation of visual field, *VFI* visual field index, *RNFLT* retinal nerve fiber layer thickness, *P P* value, *R* Spearman’s correlation coefficient

Spearman’s rank correlation was performed

Correlations significant at ***P*** **< 0.05 are bolded**

## Discussion

In this study, we found that the extent of localized RNFL angle defect is positively correlated with the visual field defect. This method not only benefits countries where OCTs are not available but also has the potential to be implemented as a new parameter for OCT in developed countries.

Several studies have already shown that RNFL thickness can be noticed earlier than visual field defect during early stages of glaucoma [18, 19]. The correlation between the structural damage of RNFL thickness and functional damage of the visual field has also been identified in multiple studies [13, 20–22]. In addition, Sommer et al. previously identified a 60% rate of structural RNFL abnormality 5 years before visual field loss, which suggested RNFL defect as a very early indicator of glaucoma [23]. The above evidences acknowledged the diagnostic value of structural RNFL abnormalities in early glaucoma, but unlike RNLF thickness, localized RNFL defect lacks a method of quantitative analysis. In this study, we showed that the measurement of RNFL angle defect around the disc proposed by Woo et al. [2] is an effective way to estimate the severity of visual field defect. It is also worth mentioning that the strength of our study depends on the reliability of visual field defect parameters after screening with the strict exclusion criteria we have imposed.

In regard to angle α, we did not find significant correlation with the presence of central scotoma. Recall that angle α is the angular width between the reference line and the proximity of RNFL defect. Hence, it makes sense that the smaller the angle α, the closer the RNFL defect is relative to the central vision and cause central visual defect. We proposed four possible explanations for the lack of correlation. Firstly, some studies suggested that RNFL thickness loss is significantly correlated with more circumferential visual field loss but is not correlated with the central visual field loss [24, 25]. Secondly, early glaucomatous optic nerve damages precede visual loss most of the time [23, 26]. Thirdly, the presence of central scotoma was identified based on 30–2 visual fields using the criteria we proposed instead of using 10–2 visual fields, of which we lack. This deficit in accurately defining central scotoma may have caused the lack of correlation. Fourthly, from a physiological standpoint, central RNFL lesion is closer to the origin of retinal vessels and may thereby receive more nutritional support, stronger cellular reinforcements, and thus develop more visual field compensation as compared to peripheral RNFL. Altogether, we believe that there may be correlation between angle α and the presence of central scotoma, but the timing of ocular examination and the definition of central scotoma may have concealed the underlying correlation in this study.

We further evaluated whether our method is comparable to that of the OCT in reflecting the severity of visual field defect through Spearman’s rank correlation test. Sectoral RNFL thickness of the OCT, which were adjusted whether it was superior temporal, inferior temporal, or combined accordingly to the RNFL defect, showed no significant correlation with visual field parameters. This suggested that our method has noninferior correlation with visual field parameters as compared to that of the OCT’s sectoral RNFL thickness. We then analyzed the correlation of global (average) RNFL thickness with the visual field parameters for the purpose of positive control and found that average RNFL thickness was significantly correlated with the visual field parameters. This suggested that in regard to the correlation with visual field parameters, our method is noninferior to OCT’s sectoral RNFL thickness and slightly inferior to the average RNFL thickness. All in all, the angular measurement of RNFLD via RNFL photograph is comparable to the average RNFL thickness of the OCT in reflecting the severity of visual field defect.

There were some limitations in our study. First, the subjectivity of angle measurements using ImageJ software limits the applicability, but this technique may be valuable if it is encompassed as one of the parameters of the OCT in the future. Second, inclusion rate is low due to high RNFL photography quality requirements. Third, the data of our participants were collected from a single medical center, which results in selection bias. Fourth, the results of visual field tests in this study were all based on 30–2 visual fields. Thus, minor visual field defects may be present under 10–2 visual fields that were undetectable via 30–2 visual fields. Fourth, diabetes and hypertension may be related to RNFLD, but we did not particularly exclude the above systemic diseases since we only seek to identify the correlation of the parameters.

## Conclusion

In summary, our study suggested that the quantification of localized RNFL defects is potentially useful for glaucoma diagnosis and that the width of RNFL defects was correlated to visual field indices in early stages of glaucoma.

## Data Availability

The datasets analysed during the current study are available upon reasonable request.

## Abbreviations

BCVA: best-corrected visual acuity
MD: mean deviation
OCT: optical coherence tomography
PSD: pattern standard deviation
RNFL: retinal nerve fiber layer
VFI: visual field index
